# Gurjun Balsam in Gonorrhœa

**Published:** 1879-04

**Authors:** 


Gurjun Balsam in Gonorrhœa.—This preparation, according to the *Bull. Gen. de Therapeutique*, has been employed with success in some of the hospitals of Paris.

The following is used by Dr. Vidal at the St. Louis Hospital:

℞.—Gurjun balsam, . . . . . ʒ j.

Gum arabic, . . . . . ʒ j.

Infusion of star anise, . . . ʒ x.

Make an emulsion. To be divided into two doses, and take immediately before meals.

Gurjun balsam is cheaper than copaiba, is said to act more rapidly, and has no unpleasant effect on the breath. —*Druggists Circular and Chemical Gazette.*

